# A digital health industry cohort across the health continuum

**DOI:** 10.1038/s41746-020-0276-9

**Published:** 2020-05-12

**Authors:** Adam B. Cohen, E. Ray Dorsey, Simon C. Mathews, David W. Bates, Kyan Safavi

**Affiliations:** 1 Health Technologies, National Health Mission Area, The Johns Hopkins University Applied Physics Lab (APL), 11100 Johns Hopkins Road, Laurel, MD 20723 USA; 2 The Johns Hopkins Hospital, Department of Neurology, 1800 Orleans Street, Baltimore, MD 21287 USA; 3 University of Rochester Medical Center, Center for Health + Technology, Saunders Research Building, 265 Crittenden Boulevard, CU 420694, Rochester, NY 14642 USA; 4 Armstrong Institute for Patient Safety and Quality, 750 E Pratt Street, 15th Floor, Baltimore, MD 21202 USA; 5 Johns Hopkins Hospital, Department of Internal Medicine, Division of Gastroenterology, 1800 Orleans Street, Baltimore, MD 21287 USA; 6 Brigham & Women’s Hospital, Department of Internal Medicine, Division of General Internal Medicine and Primary Care, 75 Francis Street, Boston, MA 02115 USA; 7 Partners Healthcare, Clinical and Quality Analysis, Information Systems, 93 Worcester Road P.O. Box 81905, Wellesley, MA 02481 USA; 8 Massachusetts General Hospital, Department of Anesthesia, 55 Fruit Street, Boston, MA 02114 USA

**Keywords:** Disease prevention, Therapeutics

## Abstract

The digital health industry has grown rapidly in the past decade. There will be few future aspects of healthcare untouched by digital health. Thus, the current status of the industry, the implications of companies’ directions and clinical focus, and their external funding are increasingly relevant to healthcare policy, regulation, research, and all healthcare stakeholders. Yet, little is known about the degree to which the digital health industry has focused on the key domains in the health continuum, including prevention, detection, and management. We performed a cross-sectional study of a US digital health industry cohort that received publicly disclosed funding from 2011–2018. We assessed the number of companies; respective funding within each part of the health continuum; and products and services by technology type, clinical indication, purchasers, and end users. In this emerging industry, most companies focused on management of disease and the minority on prevention or detection. This asymmetry, which is similar to the traditional healthcare system, represents an opportunity to focus on earlier parts of the health continuum. Patients were a common purchaser of all products, but especially prevention-focused digital health products, implying a large unmet need not yet served by the traditional healthcare system.

## Introduction

The continuum of health spans the domains of disease prevention, detection, and management^1,2^. Over the past decade, a rapidly growing cohort of >1200 companies that exclusively focus on digital health has received $33 billion in investment^3^. These companies offer products and services addressing each aspect of the health continuum. Their digital health products and services bring the promise of greater scale, efficiency, access, convenience, and patient engagement. Little is known, however, about how the digital health industry has focused on these three domains of the health continuum.

The current US healthcare system emphasizes management over prevention and detection^4,5^. In addition, chronic conditions are responsible for at least 70% of deaths and 75% of US healthcare expenditures^4^. Healthcare expenditures, too, center on management of disease, disproportionately toward acute care and sick visits, and the pharmacological, device, and services that treat acute disease states^6^. Prevention, however, accounts for about 3% of total US healthcare expenditures and <20% of National Institutes of Health funding^5,7^. Although the cost-effectiveness of current prevention-focused solutions is debated^8^, prevention represents a potentially neglected part of the health continuum. Digital health represents a new potential way to reduce disease burden cost through disease prevention, as well as other parts of the health continuum.

Few data are available regarding the digital health industry’s relative emphasis on each part of the health continuum, and the types of products and services addressing each one. Although the industry must develop products and services the healthcare system purchases, they may or may not address the actual needs of society across the health continuum.

This is a cross-sectional study from 2011 to 2018 of US-based digital health companies using the Rock Health Digital Health Funding Database maintained by Rock Health Inc^3^. Unlike other databases that include digital health companies, the Rock Health Digital Health Funding Database is curated specifically for digital health. We assessed this cohort’s focus on each aspect of the health continuum, including prevention, detection and management, the latter of which was subcategorized into treatment, monitoring, and coordination. We also evaluated the types of technologies developed, the clinical indications addressed, end users, and purchasers.

## Results

### Company characteristics

Overall, 1214 companies were identified in the database. Since the purpose of the study was to evaluate the industry’s focus on the patient health continuum, 702 (57.8%) companies met inclusion criteria based on company objectives matching at least one continuum category. These companies were focused directly on patient health while excluded companies provided other offerings (e.g., billing products, customer acquisition) (Supplementary Table 1). Supplementary Table 2 section provides a full list of company objectives and technology type descriptions.

Companies were founded between 1970 and 2018. The company business status at the end of the study period was: active 607 (86.5%); acquired 62 (8.8%); defunct 22 (3.1%); other 11 (1.6%).

### Company distribution and funding across the health continuum

**Fig. 1:**
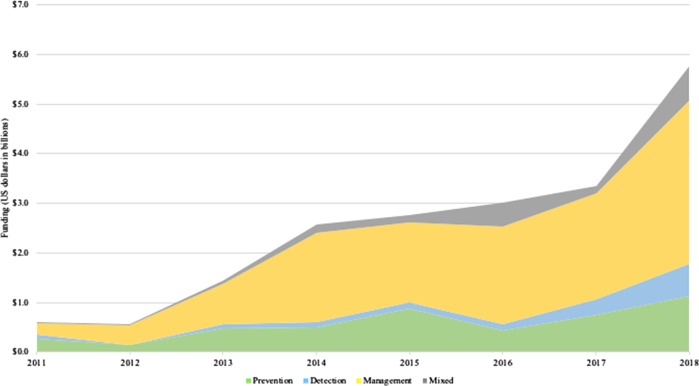
Company funding by year across the health continuum. Total funding per year for each health continuum category for companies with a singular or mixed focus among the categories.

Most digital health companies (73.2%) had a focus on management of disease, while the minority had a focus on prevention (23.8%) or detection (13.0%). The cohort received $20.8 billion of total funding from 2011 to 2018. Companies that focused singularly (only one continuum category) on disease management (64% of companies) also had the greatest total investment at $12.8 billion (61.3% of funding), compared with $4.7 billion (22.6% of funding) with a singular prevention focus (19.8% of companies), $1.5 billion (7.3% of funding) with a singular detection focus (6.8% of companies), and $1.8 billion (8.8 % of funding) with mixed focus (multiple continuum categories, 9.4% of companies). Each continuum category experienced a general growth trend in funding from 2011 to 2018, reflecting a $0.7 billion annual average growth rate for all companies (Fig. 1). Among prevention, detection, management, and mixed companies, management-focused companies experienced the most annual average growth at $0.4 billion as compared to $0.1 billion for the others.

### Company clinical indications, end users, and purchasers across the health continuum

Tables 1–3 show the analysis of the two-way combinations between company characteristics (technologies, clinical indications, end users, and purchasers) for the entire cohort (“All”). General software was the most common technology type for the entire cohort. Further, most among the cohort did not target a specific clinical indication, such as cardiology or neurologic conditions.

**Table 1:**
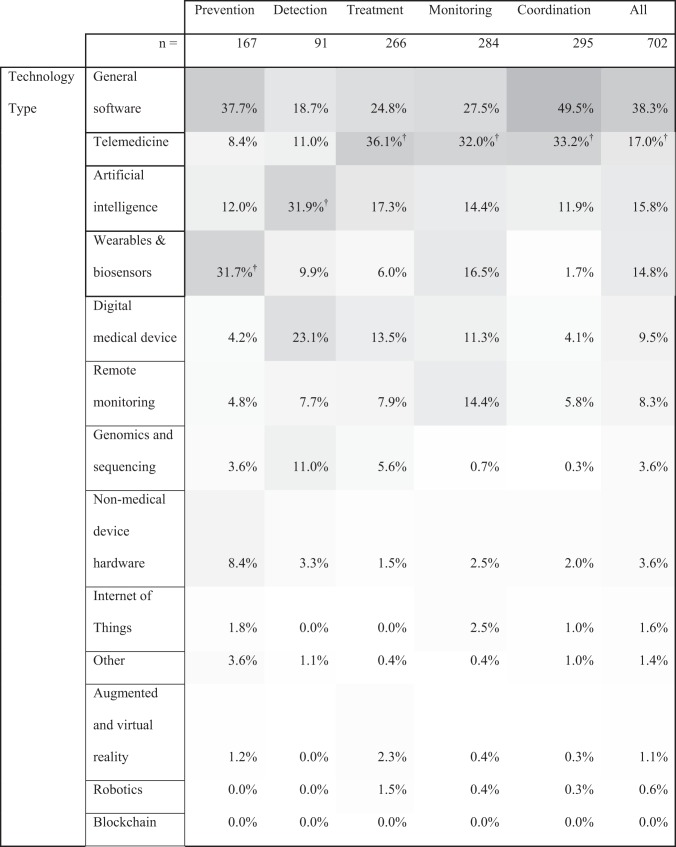
Digital health industry technology types across the health continuum.

Health continuum: the percentage of companies with each characteristic is shown for each part of the continuum. Since one company may have multiple characteristics within each category (technology type, clinical indication, end user, purchaser), rows and columns within each characteristics section add to >100%.

The shade of each cell corresponds to the combination’s frequency (i.e., a dark cell reflects a more frequent combination). All company characteristics, outside the continuum categories, were defined by Rock Health.

^†^Denotes leading combinations of a “specific” clinical indication or technology type. Here, specific refers to any technology type or clinical indication excluding “general,” “none,” “populations,” or “other”.

**Table 2:** Digital health industry clinical indications across the health continuum.

		Prevention	Detection	Treatment	Monitoring	Coordination	All
	*n*	167	91	266	284	295	702
Clinical indication	None	62.9%	18.7%	33.1%	39.8%	54.2%	46.2%
	Populations	8.4%	12.1%	17.7%	18.7%	24.1%	15.4%
	Other	5.4%	24.2%	19.2%	12.3%	8.8%	11.7%
	Neurologic	10.8%^**†**^	14.3%	7.5%	3.2%	1.4%	7.1%^**†**^
	Mental health	6.6%	3.3%	11.7%^**†**^	8.8%	6.4%^**†**^	7.0%
	Endocrine	5.4%	6.6%	6.0%	9.5%	4.7%	6.0%
	Cardiovascular	2.4%	15.4%^**†**^	3.4%	9.9%^**†**^	1.4%	5.8%
	Oncologic	0.0%	14.3%	7.1%	0.7%	1.4%	4.3%
	Women’s health	3.0%	7.7%	4.9%	3.9%	2.0%	4.1%
	Pulmonary	0.0%	1.1%	0.8%	2.8%	1.0%	1.4%

Health continuum: the percentage of companies with each characteristic is shown for each part of the continuum. Since one company may have multiple characteristics within each category (technology type, clinical indication, end user, purchaser), rows and columns within each characteristics section add to >100%.

^†^Denotes leading combinations of a “specific” Technology Type. Here, specific refers to any Technology Type excluding “general software,” or “other”.

**Table 3:**
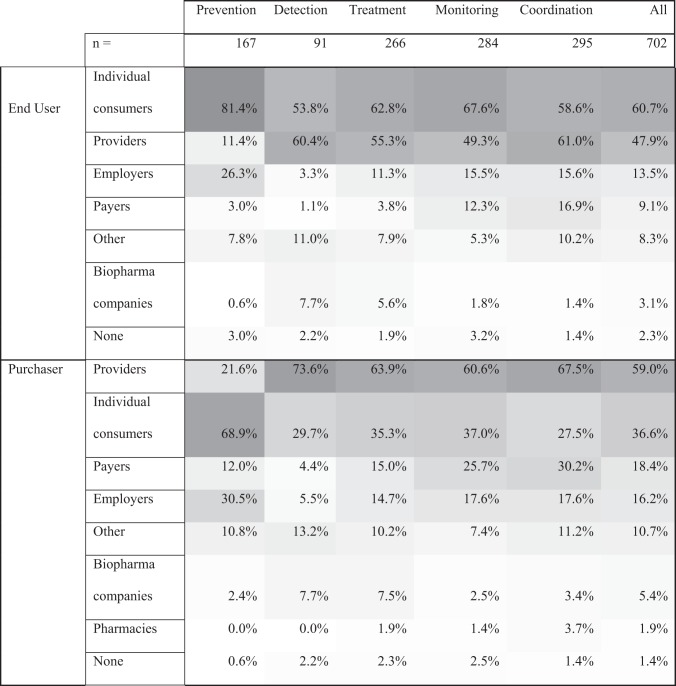
Digital health industry end users and purchasers across the health continuum.

Health continuum: the percentage of companies with each characteristic is shown for each part of the continuum. Since one company may have multiple characteristics within each category (technology type, clinical indication, end user, purchaser), rows and columns within each characteristics section add to >100%.

The shade of each cell corresponds to the combination’s frequency (i.e., a dark cell reflects a more frequent combination). All company characteristics, outside the continuum categories, were defined by Rock Health.

Tables 1–3 also show the intersection of these characteristics within each continuum category. General software was the most common technology type among companies with prevention and coordination focus; artificial intelligence among detection companies; and telemedicine for treatment and monitoring companies. Prevention-, treatment-, monitoring-, and coordination-focused companies typically did not target a specific clinical indication. Detection companies, however, most commonly targeted “other” populations, which included a range of indications such as dental and dermatologic indications.

Individual consumers or providers, as opposed to other purchaser and end-user types, were the most commonly targeted end user and purchaser for the entire cohort and across each continuum category.

Tables 1–3 show the most common “specific” technology (i.e., not general software) and clinical indication (i.e., not none, populations, or other) combinations for each continuum category. The leading such specific technologies were telemedicine, artificial intelligence, and wearables and biosensors while the leading such clinical indications were neurologic, mental health, and cardiovascular.

Table 4 shows the most frequent intersections for three key company features—clinical indication, technology type, and continuum category. The most common such combinations were companies that targeted no specific clinical indication, deployed general software, and were management-focused in the coordination domain. This combination type was more than twice as common as any other type. All such three-way combinations are shown in the Supplementary Table 3.

**Table 4:**
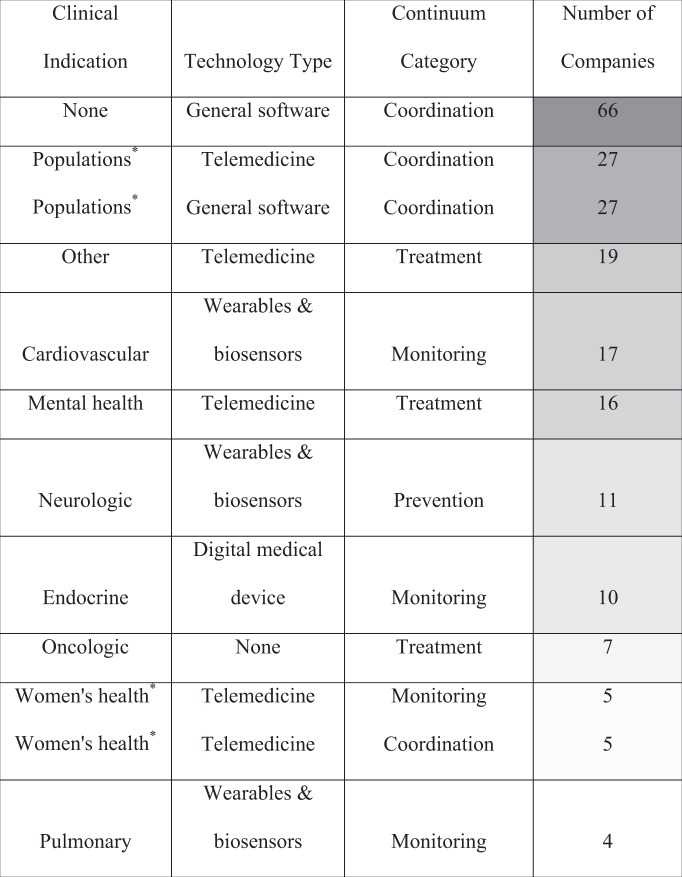
Top clinical indication/technology type pairings across health continuum.

The shade of each cell corresponds to the combination’s frequency (i.e., a dark cell reflects a more frequent combination). All company characteristics, outside the continuum categories, were defined by Rock Health.

*Only for the “populations” and “women’s health” clinical indications were there ties for top pairings; both sets of ties are shown in the table.

Of note, blockchain, robotics, and augmented and virtual reality technologies, and women’s health-focused companies, despite being highly discussed in the media and literature, were among the least common areas of technology or clinical indication focus for the entire cohort and for most of the continuum categories. Pulmonary-focused companies were the least common clinical indication for the entire cohort and across all continuum categories.

## Discussion

In terms of both the number of companies and total funding dollars, this large digital health industry cohort has focused on disease management over disease prevention or detection. Overall, between 2011 and 2018, we found that less than one-quarter of digital health investment dollars went to companies singularly focused on disease prevention and even fewer on disease detection. Similarly, 23.8% and 13% of companies focused on prevention or diagnosis, respectively, while 73.2% focused on management.

This cohort’s relative lack of focus on disease prevention, and relatively accelerated growth of management-focused companies, is likely multifactorial. The relative lack of focus on prevention is similar to historical estimates of the total expenditure in healthcare on prevention services^5^. The outsized focus on management of disease may be related to a less robust marketplace for prevention- and detection-focused products. US-based healthcare reimbursement, which is mainly fee-for-service, largely pays providers for management activities. A different pattern may be seen in other countries with more value-based care and different incentive structures. Further a different pattern may be seen if the US were to increasingly adopt new reimbursement models, such as accountable care organizations, which are currently limited in scope.

Reimbursement of disease prevention, however, is far less clear under the US-based healthcare reimbursement structure. Participating payers may not receive financial benefits since younger beneficiaries may change insurers. Only beginning in 2005 did Medicare begin to cover prevention visits, but beneficiary participation remains low^9,10^. To determine coverage, Medicare performs cost-effectiveness analyses for preventive services but not treatment services^5^. Lifestyle modification programs, for example, have been shown to be more effective than metformin to prevent diabetes, but only the latter is broadly covered by US insurers^5,11^. Given the lack of reimbursement for prevention-focused activities, digital health companies may be focusing on offerings that fit into the current reimbursement model. If US healthcare shifts to a value-based system, however, prevention- and detection-focused digital health technologies may find a more robust market in digital health.

Digital health companies offering technologies that reduce care inefficiencies, cost, and administrative burden are poised to address to support value-based care in the US^12,13^. Such companies could reduce the $760 to $935 billion in annual waste, estimated to account for 25% of total care spending^14^. Other digital companies enabling value-based care offer care coordination functions, provide access to behavioral health, and address certain social determinants of health^12,13^. The traditional healthcare system has struggled with all of these areas. Further, care team satisfaction and experience issues, which may account for at least $4.6 billion in annual costs^15^, is an emerging digital health focus and another potential enabler of value-based care^12,13^.

Additionally, effective disease prevention strategies may reside outside the typical boundaries of healthcare, which rely upon patient-provider interactions within clinics and hospitals^16^. Strategies appealing directly to the patient may be more impactful than those involving the traditional healthcare system. Major health organizations and governing bodies, including the World Health Organization, have cited “Health Promotion” as a central goal to improve health and minimize patients’ interactions with the healthcare system through disease prevention^17^. Digital health products and services represent such approaches to care outside traditional healthcare interactions, potentially at much lower cost. We found that when digital health companies focused on prevention, they most commonly targeted patients both as end users and purchasers. If the most effective prevention strategies directly engage patients without requiring traditional healthcare setting interactions, digital health may shift toward prevention. Patients already demand and consume products not provided by traditional healthcare, including the wide range of complementary and alternative medicine products and services. Future studies should further explore the mismatch between patient needs, what traditional healthcare provides, and what gaps can be addressed by digital health.

Although much of the conversation in digital health centers on emerging, specific differentiating technologies (e.g., artificial intelligence, blockchain), we found that the most common digital health companies deployed only general software and not these differentiating technologies. Further, companies most commonly had no particular clinical indication focus, indicating an industry-wide trend not targeting specific conditions or clinical problems, which has been reported previously^18^. This approach potentially limits the ability to impact specific high-burden conditions since companies’ may not be structured to explicitly address them.

We also found that patients were a common purchaser of digital health products and services; they were the lead purchaser among prevention digital health products and services. This implies patients demand and consume health services not provided by the traditional healthcare system, which suggests a large unmet need.

Wearables and biosensors were the most commonly used specific technology types among prevention companies. Given the wide array of sensor types (e.g., accelerometers, cameras) and form factors (e.g., headbands, watches)^19^, many conditions and digital biomarkers are being investigated, potentially paving a road toward increased condition-specific prevention digital health technologies. These technologies, first validated to detect or monitor a specific condition, may be repurposed to prevent that condition by identifying early warning signs. For example, in the past two years, the US Food and Drug Administration cleared medical devices such as wearable glucose monitors that targeted diabetics^20,21^. As such technologies become less invasive, they could be validated in healthy people at risk of developing diabetes to identify glucose perturbations that predict disease onset, thereby enabling targeted lifestyle and medication prevention strategies.

Neurologic conditions represented the most common condition targeted for prevention. Randomized-controlled trials of digital health products and services have predominantly evaluated management- and not prevention-focused digital health technologies for neurological conditions^22^. In the peer-reviewed literature, prevention-focused companies targeting cognitive cognitions (through smartphone-based cognitive games for which data are limited)^23^ and sleep (through varied smartphone- and wearables/biosensors-based approaches for which data are stronger for digital cognitive behavioral therapy for insomnia^24^).

Although we identified no prevention-focused companies targeting oncology or pulmonary conditions, this may have been an artifact of our company characterization scheme. Digital health technologies that targeted modifiable risk factors such as smoking cessation, for example, would impact varied pulmonary diseases, but would not have been characterized as pulmonary-focused in our analysis. Further, one group reported that over 40% of all incident cancers and cancer deaths were attributable to modifiable risk factors such as cigarette smoking, excess body weight, and alcohol intake^25^. Exemplar risk factor-focused digital health technologies include mobile-based cognitive behavioral therapy for smoking cessation or text message- or interactive mobile app-based recommendations tailored to the patient’s risk factor^26^. The recommendation-based approaches can be paired with various technologies to track measures related to exercise^26^. Nevertheless, despite this indirect potential focus on oncology or pulmonary conditions, there likely exist opportunities to target these potential digital health gaps given the high burden of cancer, chronic obstructive pulmonary disease, and asthma.

More than any other continuum category, detection-focused digital health companies built artificial intelligence products. Artificial intelligence has potential utility in every aspect of healthcare^27^, but machine-assisted image interpretation (radiologic, pathologic, optical) applications have been the most studied^28^. Although artificial intelligence performance has matched or exceeded clinician performance in controlled study environments and the US Food and Drug Administration (FDA) approvals for these algorithms are accelerating, few studies have prospectively measured impact in real world clinical environments^28^. At present, these applications assist clinicians and thus, consistent with our findings, providers are the most commonly targeted end users and purchasers.

Cardiovascular, oncologic, and neurologic conditions were the most common indications among detection-focused companies. Cardiovascular disease detection has been studied for many conditions using diverse sensor data (e.g., electrocardiogram, optical coherence tomography)^29,30^. Several companies use wearables that capture heart rate or rhythm to detect cardiologic conditions like atrial fibrillation, an emerging area for which best practices have not been established^31,32^. Some claim that similar data can detect non-cardiac conditions like diabetes mellitus, hypertension, hyperlipidemia, and sleep apnea^33^.

Clinicians represent the primary audience for most detection-focused technologies. As such, these tools may be incorporated into clinical practice. Hospital systems and their clinicians unaccustomed to these emerging tools must learn how to compare, use, select, and integrate these tools into care, while policy makers, researchers, and medical societies must continue to study the impact of these technologies in practice.

Telemedicine represented the most common specific technology used by the digital health industry to impact care management. This trend existed across all management categories, and is aligned with other reports demonstrating that telemedicine companies are among the most highly funded in the digital health industry^34^. Such a trend will likely continue as telemedicine reimbursement parity (as compared to routine clinical counters) increases.

Despite the high level of company activity in telemedicine we observed, telemedicine remains a low overall proportion of actual care, including rural Medicare and Veterans Administration beneficiaries, despite the relatively common (34%) lifetime exposure to telemedicine for a general consumer population^35^. Taken together, this suggests this part of the industry has yet to generate a broad-based impact on patient care management or efficiency^36^. While the case for broader impact remains under investigation, the impact of certain condition-specific use cases has been clearly demonstrated. Acute stroke, for example (telestroke), has been well-studied and demonstrated to be safe and effective^37^.

We found that mental health conditions were the most common specific indication for all management-focused companies—and the most common pairings among mental health-focused technologies were telemedicine-management companies. Well-studied mental health management technologies include telemedicine services and cognitive behavioral therapy as a digital therapeutic (i.e., digital medical device), which have varying quality data to support effectiveness^38,39^. Oncologic, pulmonary, and pediatric areas have been less of a focus among management companies and may represent an opportunity for the digital health industry.

In this study, the second most common specific indication was cardiovascular for management companies focused on monitoring. Currently available cardiovascular digital health monitoring technologies mainly target arrhythmias through a smartphone adapter, electrocardiogram patch, or smart band paired with a smart watch for which early results are promising in select populations like those with or at risk for atrial fibrillation patients^31,40,41^. Given the high burden of rehospitalization, congestive heart failure digital monitoring strategies primarily focus on preventing readmission by tracking modifiable factors associated with clinical decline, such as blood pressure and weight^42^. Managing modifiable cardiovascular risk factors (weight, body mass index, blood pressure, lipid levels, smoking, diet, physical activity) represents another common digital health approach through monitoring and management-focused products^41,43^. A 2015 meta-analysis of 51 digital health intervention studies showed improvements in weight, body mass index, and Framingham risk scores, but not blood pressure^44^.

This study had several limitations. Our cohort is not inclusive of all digital health companies from 2011 to 2018. For example, companies that develop digital health products but were not exclusively dedicated to digital health (or healthcare) were not included. Some of these companies, particularly large ones, produce some of the best-selling^45^ and widely distributed digital health products. Since our database drew from disclosed US deals from 2011 to 2018, we did not include digital health companies who did not disclose US deals or did so outside 2011–2018.

This is a potential source of bias in our findings. For example, we may miss the contribution of smaller, early stage companies potentially focusing on prevention of disease and thus changing the direction of the industry. In addition, international companies may be more apt to generate prevention-focused digital health products given differences in reimbursement structure and policy in the healthcare systems of other countries. Since our dataset contained only US companies, we could not assess companies outside the US. A future analysis of companies outside the US will be of great interest. Compared with the US, countries with different care models and incentive structures, such as single-payer systems, may foster digital health environments that focus on other portions of the care continuum, such as prevention. Other potential company characteristics were not captured in our assessment, such as whether or not the company had regulatory approvals or published efficacy data, but will be useful for future assessments.

The dataset used, although not representative of all digital health companies, is the most comprehensive publicly available digital health company set available to our knowledge. Other databases also contain digital health companies and can be used to assess the industry. To our knowledge, such other databases do not offer company characteristics pertinent to the questions posed in this study (e.g., clinical indication, technology type). Further, they were less amenable to industry-wide digital health analyses since no distinctions were made between digital health and the broader field of healthcare. Further, the digital health definition used by Rock Health to include companies in the database was similar to our definition, which is based on that of the FDA. Other potential company characteristics were not captured in our assessment, such as whether or not the company has regulatory approvals or published efficacy data and to what degree, but will be useful for future assessments.

We attributed company objectives to specific continuum categories. This was based on Rock Health’s definitions of these company objectives and consensus among the authors. Our attributions might differ from those attributed by another set of authors. Further, the continuum-company objectives may oversimplify a particular company’s actual focus, which may extend beyond the continuum category labels we assigned.

It is also possible that the other company characterizations do not fully capture each attribution type, such as company objective, technology type, and clinical indication for each company assessed. A large portion of the companies were general software in technology type, which mainly represent software services and mobile apps. Future assessments should tease out the subtypes of such products. Clinical indication categories were broad (e.g., neurologic) and further studies could also assess specific conditions (e.g., stroke, Parkinson disease).

Further, although companies addressing a risk factor like smoking, for example, could have been assigned a clinical indication reflecting conditions it could prevent or manage (e.g., pulmonary, cardiovascular diseases), the company may have been assigned another clinical indication (e.g., substance abuse disorder in mental health). Although we assigned each company at least one main continuum category attribution, one company could have more than one attribution, which allowed a potentially more accurate reflection of the companies range across the continuum.

Our results describe the focus of digital health but not the impact. Leading privately funded digital health companies have not produced substantial impact on disease burden or cost as measured by reports on their products in peer-reviewed publications^18^. In this study, however, we did not report on the effect of any of these companies’ products and services within any continuum category. The relative size of each continuum category by number of companies or total funding did not necessarily equate to effectiveness. Even though prevention companies were the smallest in number and least funded, for example, some or many of them could have had great impact.

In the emerging digital health industry, we found an outsized focus on management of disease compared to prevention and detection. This represents an opportunity to build an environment that focuses on earlier parts of the health continuum. After general software, wearables, artificial intelligence, and telemedicine were the most common specific technology types across the sector. As a result, training and educating the physician community on how best to use these technologies will become increasingly important. Given that most technologies targeted clinicians and patients directly, they require a similarly rigorous validation as traditional healthcare solutions to ensure their use improves outcomes and cost-effectiveness. Lastly, patients were a common purchaser of digital health products and services, and prevention-focused ones in particular. Thus, a large unmet need likely exists, which is not yet fulfilled by the traditional healthcare system.

## Methods

### Definitions

The definition and categorization of digital health that we used align with those used by the FDA. Digital health represents technologies that enable consumers to make informed decisions about health across prevention, diagnosis, and management of disease without requiring traditional care settings^46^. Categories of digital health included in this study are also specified by the FDA^47,48^.

### Database and data

#### Companies, products, and services

We performed a cross-sectional analysis using the Digital Health Funding Database maintained by Rock Health Inc^3^. Company characteristics assessed were founding year, company status (e.g., active, acquired, defunct), and total amount of publicly disclosed funding.

The database is a repository of US-based digital health companies that disclosed at least $2 million in funding from investors from 2011 to 2018^3^. Companies with total funding under $2 million were excluded as such companies are not consistently filed in the public domain.

Digital health companies were defined as those that build and sell healthcare-focused technologies and services paired with these technologies, such as physician consultation through a telemedicine or remote monitoring platform.

### Variables

Company characteristics (Supplementary Table 2) of company products and services included (1) company objective (i.e., value proposition) of the product or service, (2) technology type of the company’s product, (3) clinical indication the company targets, (4) the end-user type (those who ultimately use the product or service), and (5) purchaser type. A single company could have multiple characteristics. Company characteristics were assigned by data abstractors from Rock Health based on reviews of multiple online sources using publicly available information, including public filings, press releases, industry databases, and company websites. The reviews are conducted and findings are updated on a quarterly basis.

### Technology types

Technology types included artificial intelligence, augmented and virtual reality, digital medical device, genomics and sequencing, internet of things, non-medical device hardware, remote monitoring, telemedicine, wearables and biosensors, other, and general software, which included varied software services and mobile apps. Supplementary Table 2 includes definitions for each of the characteristics for company objectives and technology types.

### Clinical indications

The authors grouped clinical indications into ten groups: neurologic (included sleep), mental health (included substance use disorders), cardiovascular, pulmonary, oncologic, endocrine, women’s health, and other (included dental and dermatologic). The penultimate group, “populations”, referred to companies targeting a broad swath of a population or a medical field outside the aforementioned indications. These included pediatrics, surgery, aging, and underserved populations. The last group, “none”, referred to a company that did not target any particular clinical indication.

### Health continuum

Prevention products and services were defined as those aiming to reduce the incidence of disease by tracking or promoting healthy behaviors (e.g., a digital fitness or activity tracker that measures physical movement). Detection products and services were defined as those aiming to diagnose medical conditions and diseases (e.g., wearable that measures electrocardiogram tracings to identify atrial fibrillation). Management products and services were divided into three aspects of care: treatment, monitoring, and care coordination. Treatment products and services included those that intervened on diagnosed conditions or enabled interventions and recommendations to be rendered in the care of a patient. Monitoring products and services tracked progress or identified exacerbations of diagnosed conditions. Coordination products and services encompassed provider activities to care for patients.

### Company inclusion criteria and continuum category attribution

Companies were included if they provided patient care-focused products or services in one or more of the following company objective categories defined by Rock Health (Supplementary Table 2). Each company objective was then assigned to one or more continuum of health categories using the schema in Table 5. Companies were then excluded if their objective was not directly related to patient care and did not fit into the health continuum concept. Such examples included non-clinical workflow, data infrastructure, and customer acquisition and relationship management.

**Table 5:** Continuum of health category assignments based on company objectives.

	Continuum categories				
	Prevention	Detection	Management		
			Treatment	Monitoring	Coordination
Objectives					
Fitness and wellness	✓				
Prevention of disease	✓				
Diagnosis of disease		✓			
Treatment of disease			✓		
Clinical decision support and precision medicine			✓		
On-demand healthcare services			✓	✓	✓
Population health management				✓	✓
Monitoring of disease				✓	
Care coordination					✓
Patient adherence					✓

Company objectives were defined by Rock Health.

### Reporting summary

Further information on experimental design is available in the Nature Research Reporting Summary linked to this article.

## Supplementary information

Supplementary Information

Reporting Summary

## Data Availability

The datasets analyzed within the Digital Health Funding Database are available by request through Rock Health at https://rockhealth.com/data/funding-raw-data/^3^. The datasets were used with persmission from Rock Health for the current study, and are also publicly available. Data are also available from the authors upon reasonable request and with permission of Rock Health.
