# Supplementary material for: A digital health industry cohort across the health continuum
Source: NPJ Digit Med. 2020 May 12;3:68. doi: 10.1038/s41746-020-0276-9 (PMC7217869; doi:10.1038/s41746-020-0276-9)
Supplement: Supplementary file 1 — Supplementary Information [file 41746_2020_276_MOESM1_ESM.pdf]

Contents

Supplementary Table 1. Company objectives for the 512 excluded digital health companies.

Supplementary Table 2. Definitions of digital health company objective (including the included and excluded categories) and technology type characteristics.

Supplementary Table 3. Activity at each intersection of clinical indication, technology type, and continuum category (number of companies per cell).

Supplementary Table 1. Company objectives for the excluded digital health companies **(**n=512).

Company objective	Percent of excluded companies (n)
Non-clinical workflow	22.1% (113)
Consumer health information	15.2% (78)
Research & development catalyst	14.1% (72)
Clinical workflow	11.9% (61)
Marketplace	8.8% (45)
Data infrastructure & interoperability	7.8% (40)
Health benefits administration	7.2% (37)
Customer acquisition & relationship management	6.8% (35)
Medical reference / education	3.1% (16)
Other	2.9% (15)

Supplementary Table 2. Definitions of digital health company objective (including the included and excluded categories) and technology type characteristics

Category	Characteristic	Definition
Objective	Consumer health information	Consumer health information companies empower consumers to better understand their own health, general healthcare concepts, and the healthcare system. The delivery of this objective often takes the form of content or other informational or educational tools.
	Marketplace	Marketplaces deliver value to at least two participants, buyers and sellers. The value of a marketplace is delivered to both/all participating groups. The buyer, customer, and consumer derive value by virtue of being able to comparison shop, discover prices, and more smoothly access resources and services. The sellers or service providers derive value by virtue of being able to reach buyers or customers more readily.
	Non-clinical workflow	Management of administrative, non-clinical operations in a provider setting such as inventory, scheduling, compliance, billing and revenue cycle, supply chain, and other activities necessary to run a hospital which generally take place outside of the patient

	exam room. Note: For the purposes of this categorization, a pharmacy is considered a "provider setting."
Clinical workflow	Enhancing the clinical workflow of doctors and other providers of patient care (e.g., placing orders, documentation into the electronic health record). Clinical workflow often takes place in the exam room or otherwise in the course of doctor- or provider-patient interactions. Note: Clinical Decision Support tools are, inherently a kind of "Clinical workflow"; however, when a company received the Clinical Decision Support label it did not also receive the "Clinical workflow" label.
Clinical decision support & precision medicine	Providing timely information or alerts to a healthcare provider (e.g. doctor, nurse, or allied health care professional), usually at the point of care, to help inform decisions about a patient's care, or tailoring the prevention, management, or treatment of disease to an individual patient. Note: Includes treatment planning tools (i.e., tools used to focus or refine the plan to deliver an intervention to be used after the decision to pursue that intervention is made. For example, dose calculation for radiation oncology or onco-genomics testing decision may be guided by such tools to help a physician and patient plan a regimen of targeted therapies.

Data infrastructure & interoperability	Enabling backend connectivity across systems and or optimizing data for use, which may include: aggregating or harmonizing data; enabling intercommunication or interoperability between systems; infrastructure for data analysis and visualization.
Customer acquisition & relationship management	Support for attracting, retaining, and managing patients or other target customers.
Health benefits administration	Optimizes selection or delivery of health benefits by employers. Enables employers to track value (of benefits). Note: Delivery of insurance coverage to individuals is not included in this category.
Population health management	Companies in this category enable entities (e.g., health systems, self-insured employers, payers) to reduce the total costs and improve health outcomes of a given patient population over time and thus manage the financial risk of that population (e.g. leveraging clinical data integration, network design, risk stratification, early intervention, readmission prevention). This category includes services that support the creation and/or delivery of value-based payment models.

Research & development catalyst	Enable the administration and execution of research and development processes (e.g., drug discovery, clinical trial management).
Care coordination	The management and communication of patient care across settings and providers/caregivers. Companies in this category particularly enable and/or improve handoffs between those who provide care.
Fitness & wellness	General health maintenance and promotion, including fitness, nutrition, and sleep, where the prevention of illness does not associate with a specific condition. Note: While companies enabling a healthy lifestyle inherently seek to prevent consumers from developing diseases, the "Prevention of disease" category distinguishes companies that target prevention of a particular condition (instead of using both "Fitness & wellness" and "Prevention of disease").
Prevention of disease	Companies for which the mandate is to prevent consumers from contracting a specific clinical condition (e.g. diabetes).
Treatment of disease	Delivers or enables the treatment or management of a specific clinical condition (e.g. substance use). Typically, "Treatment of disease" solutions are associated with a specific clinical condition. Note: "Clinical decision support" tools are excluded from this

		category. Note: Tools that enable adherence to a prescribed regimen are not included in the "Treatment of disease" objective. These fall within the "Patient adherence" objective. Note: Pure telemedicine companies (i.e., companies that offer enabling telemedicine technology without respect to the specific treatments being offered) are excluded from this category.
	Diagnosis of disease	Enables the diagnosis of a specific clinical condition (e.g. diabetes).
	Monitoring of disease	Enables the monitoring of a specific clinical condition (e.g. diabetes). Must be monitoring a biometric (not patient adherence).
	On-demand healthcare services	The delivery of immediate, or near real-time, healthcare services (e.g. physician house calls, telemedicine)
	Patient adherence	Allows for and supports patients in adhering to their prescribed medication regimen, discharge instructions, or care plan.
	Medical reference / education	Delivers information to or enables teaching of providers by delivering content, training, or other learning tools.
	Other	Companies that do not fit any of the aforementioned objectives.

Technology Type	Artificial intelligence	The use of data, algorithms and machine learning techniques (e.g. designed with the ability to learn without explicit programming) to identify the likelihood of future outcomes based on historical data or perform tasks that normally require human intelligence. Note: Artificial intelligence companies' offerings may include (but are not limited to) visual perception, speech recognition, decision-making, and translation / natural language processing, artificial neural networks, and chatbots.
	Wearables and biosensors	Wearable or accessory devices (not necessarily worn) that detect specific biometrics and are intended for consumers to track themselves
	Genomics & sequencing	Hardware and software technologies that sequence, assemble, call variants, and otherwise analyze sequencing data (e.g. sequencing on a chip with data aggregation).
	Telemedicine	Technologies that enable the delivery of healthcare services (synchronous or asynchronous) from a person (not a chat-bot, automated symptom checker, etc) when the service provider is in a different physical location from the service recipient. Note: Companies in this category may offer virtual visits via telephone, digital imaging, videoconferencing, and coaching.

	Remote monitoring	Technologies that enable the tracking and monitoring (of one person by another/others/machine) of information when a person is not in the presence of a caregiver or provider. Requires that the information is being transmitted to another person (not self-monitoring). Enables caregiving in lower cost site of care. Also enables non-medical monitoring (typically by family/caregivers in a non-medical setting).
	Augmented and virtual reality	Technology that superimposes a computer-generated image on a user's view of the real world, thus providing a composite view or simulates an artificial environment that may interact within a seemingly real or physical way by a person.
	Internet of Things	Connected sensors that measure the physical environment (not biometrics), creating a network of "things" that appropriately interact with one another toward common function(s). Note: Sensors that collect measures of human activity are tracked in the "wearables and biosensors" category.
	Non-medical device hardware	Connected equipment or hardware designated for professional or at-home use that does not require FDA approval. Note: This category does NOT include wearables and biosensors (which are included in the “wearables and biosensors" category).
	Robotics	Use of robots to deliver healthcare services

Supplementary Table 3. Activity at each intersection of clinical indication, technology type, and continuum category (number of companies per cell).

	Continuum Category[2]	Wearables & biosensors	Telemedicine	Genomics and sequencing	Artificial intelligence	Digital medical device	Internet of Things	Non-medical device hardware	Robotics	Blockchain	Remote monitoring	Augmented and virtual reality	Other	General
Cardiovascular	P	3	1	0	2	1	0	0	0	0	1	0	0	0
	D	2	0	1	4	2	0	0	0	0	4	0	1	3
	T	2	3	0	3	3	0	0	0	0	3	0	0	1
	M	17†	1	0	4	6	0	0	0	0	10	0	0	2
	C	0	1	0	0	0	0	0	0	0	1	0	0	2
Endocrine	P	4	5	0	3	1	0	0	0	0	0	0	0	1
	D	1	3	0	2	2	0	0	0	0	2	0	0	0
	T	2	7	0	4	5	0	0	0	0	2	0	0	2
	M	6	4	0	3	10†	0	1	0	0	6	0	0	2
	C	0	6	0	2	5	0	0	0	0	4	0	0	1
Mental health	P	0	2	0	1	0	0	0	0	0	0	0	0	8
	D	0	1	0	1	0	0	0	0	0	0	0	0	1
	T	1	16†	0	6	4	0	0	0	0	2	0	1	6
	M	1	12	0	3	3	0	0	0	0	2	0	0	2
	C	0	12	0	1	0	0	0	0	0	0	0	0	2

Neurologic	P	11†	1	0	1	0	0	0	0	0	2	0	1	4
	D	1	1	0	5	5	0	1	0	0	1	0	0	2
	T	4	2	1	5	6	0	1	1	0	3	1	0	2
	M	4	2	0	4	2	0	0	0	0	2	0	0	0
	C	0	2	0	1	0	0	0	0	0	1	0	0	1
Oncologic	P	0	0	0	0	0	0	0	0	0	0	0	0	0
	D	2	0	3	5	3	0	0	0	0	0	0	0	2
	T	0	1	5	6†	2	0	0	1	0	0	0	0	7
	M	0	1	0	0	0	0	0	0	0	0	0	0	0
	C	0	1	0	0	0	0	0	0	0	0	0	0	2
Pulmonary	P	0	0	0	0	0	0	0	0	0	0	0	0	0
	D	0	0	0	0	1	0	0	0	0	1	0	0	0
	T	0	0	0	0	1	0	0	0	0	2	0	0	0
	M	4	0	0	0	3	1	1	0	0	2	0	0	1
	C	0	1	0	0	2	1	1	0	0	2	0	0	0
Women's health	P	1	1	0	1	1	0	0	0	0	0	0	0	1
	D	0	0	2	1	4	0	0	0	0	0	0	0	1
	T	2	4	1	1	3	0	0	0	0	1	0	0	2
	M	1	5†	1	2	1	0	0	0	0	1	0	0	2
	C	0	5†	0	1	0	0	0	0	0	0	0	0	1
Other	P	2	1	0	1	3	0	2	0	0	0	0	0	2
	D	1	5	3	1	7	0	3	0	0	2	0	0	5
	T	4	19†	3	1	15	0	0	0	0	3	2	0	10
	M	8	15	0	3	5	0	0	0	0	5	0	0	8
	C	0	16	0	0	2	0	0	0	0	2	0	0	8

Populations[1]	P	4	1	3	3	0	0	1	0	0	3	0	0	3
	D	1	2	2	3	1	0	1	0	0	0	0	0	2
	T	2	24	1	7	0	0	1	2	0	7	2	0	9
	M	6	24	1	6	1	2	3	1	0	9	1	0	8
	C	2	27†	0	6	1	1	2	1	0	8	1	0	27
None[1]	P	30	5	3	11	1	3	11	0	0	3	2	5	46
	D	1	0	2	9	2	0	0	0	0	0	0	0	4
	T	3	30	5	16	3	0	2	0	0	4	1	0	34
	M	8	29	1	5	5	3	2	0	0	10	0	0	17
	C	2	33†	1	8	3	0	3	0	0	3	0	2	66

[1]“Populations” and “none” are shown in a separate sub-table with separate frequency-color mappings to allow better visualization between the two sub-tables because these two clinical indications contain extreme outliers as compared to the others.

[2]Continuum categories: P = prevention, D = detection, T = treatment, M = monitoring, C = coordination

†Denotes leading combinations of a “specific” Clinical Indication or Technology Type. Here, specific refers to any Technology Type or Clinical Indication excluding “general,” “none,” “populations,” or “other.”

Note: The shade of each cell corresponds to the combination’s frequency (i.e. a dark cell reflects a more frequent combination). All company characteristics, outside the continuum categories, were defined by Rock Health.
